# Impact of Prefabricated, Universal 2-Piece, and CAD/CAM-Milled Fiber Posts on Bond Strength and Microhardness of a Self-Adhesive Resin Cement in Widened Root Canals

**DOI:** 10.4317/jced.63143

**Published:** 2025-09-01

**Authors:** Rodrigo Stadler Alessi, Giovana Mongruel Gomes, João Carlos Gomes

**Affiliations:** 1 DDS, MS. PhD student, Postgraduate Program in Restorative Dentistry, Department of Restorative Dentistry, State University of Ponta Grossa (UEPG), Ponta Grossa, Paraná, Brazil; 2 DDS, MS, PhD. Professor, Department of Restorative Dentistry, School of Dentistry, State University of Ponta Grossa (UEPG), Ponta Grossa, Paraná, Brazil; 3 DDS, MS, PhD. Professor, Department of Restorative Dentistry, School of Dentistry, State University of Ponta Grossa (UEPG), Ponta Grossa, Paraná, Brazil

## Abstract

**Background:**

The objective of this study was to assess the push-out bond strength (PBS) of conventional prefabricated, universal 2-piece, and CAD/CAM-milled fiber posts cemented in root canals using a self-adhesive resin cement, as well as the Vickers microhardness (VHN) of the resin cement.

**Material and Methods:**

Thirty human uniradicular premolars roots were endodontically treated and divided into three groups (n = 10): conventional prefabricated fiber posts (PFPs), universal fiber posts (UFPs), and CAD/CAM-milled fiber posts (MFPs). After luting procedures using RelyX U200 (Solventum), six specimens were obtained of each root (two slices from each root third: cervical, middle, and apical). The first slices of each root region were subjected to PBS, and the second slices were subjected to VHN analysis. Data from the PBS and VHN tests were analyzed using two-way analysis of variance (ANOVA; post type vs. root region) and Tukey’s test (α = 0.05).

**Results:**

Regarding the PBS, MFPs and UFPs demonstrated statistically superior performance than PFPs (*p* < 0.001). Among the root regions, the cervical third exhibited the highest values, whereas the apical third showed the lowest (*p* < 0.001). Regarding VHN, PFPs and UFPs exhibited statistically superior values compared with MFPs (*p* < 0.001). The cervical third of the root displayed the highest VHN values, whereas the apical third presented the lowest (*p* < 0.001).

**Conclusions:**

CAD/CAM-milled and universal 2-piece fiber posts may be a better alternative for restoring widened root canals.

** Key words:**Resin Cements, CAD-CAM, Post and Core Technique, Hardness Tests, Root Canal Preparation.

## Introduction

Intraradicular posts are often necessary for retention in core or coronal restorations of teeth that have undergone endodontic treatment and suffered significant structural loss [1]. Glass fiber posts (GFPs) are frequently recommended due to their mechanical properties that mimic those of dentin, such as a good elastic modulus. These properties reduce tension in the root canal and diminish the probability of radicular fractures [2,3]. Moreover, their chemical properties correspond with those of resin cements commonly employed in adhesive procedures [4].

Prefabricated fiber posts (PFPs) in widened root canals require careful luting because they do not precisely fit into root canal preparations, and the resin cement layer is often thick and has bubbles or voids, increasing the likelihood of failures and making it difficult to guarantee proper retention and stability [3,5,6]. Thus, the customization of GFPs using resin composites is recommended in these circumstances [3,6]. This customization ensures that the fiber post is more precisely adapted to the root canal, improving the mechanical qualities of the posts [7,8].

The universal 2-piece fiber post (UFP), including a fiber post and a universal sleeve, is a recently proposed revolutionary solution. The sleeve and fiber post are composed of identical material. The manufacturer asserts that UFPs are suiTable for application in root canals of diverse dimensions, including larger ones, and that they ensure better adaptation and mechanical attachment in the cervical region of the root canal. Key characteristics include universality, anatomical design, strong retentiveness, careful preparation, and a low risk of root fracture [9]. Initial experiments comparing UFPs to PFPs and relined fiber posts using composite resins have produced promising outcomes. These studies have shown superior or equivalent root canal adaptation, enhanced frictional retention, and the achievement of a minimal cement layer thickness [10-12].

Technological improvements, such as computer-aided design and computer-aided manufacturing (CAD/CAM), have enabled the manufacture of post-and-core units in a single piece. This innovation reduces the number of interfaces between the fiber post and the resin composite core, thereby minimizing the risk of structural failure [13]. CAD/CAM-milled post-and-cores (MFPs) exhibit improved biomechanical behavior due to their excellent adaptation to the root canal, promoting increased frictional retention and a thin cement layer [14-17].

The adhesive cementation of fiber posts into radicular dentin is an extremely delicate process that presents significant challenges for clinicians due to the various operative stages that must be completed before the resin cement fully polymerizes [18]. Resin cements with dual- or self-cure properties are the best choices for luting agents to counteract mild attenuation brought on by root canal depth and the post [19]. The chemical curing technique of dual-cure resin cements enables polymerization to continue in regions not exposed to light [20]. The processes of light and chemical curing are complementary and separate from one another [21]. Certain dual-cure resin composites have been reported to be slower and less effective, or nearly ineffective, despite the effectiveness of chemical curing in areas that are not exposed to light [22].

The degree of conversion attained by a composite influences mechanical qualities and susceptibility to deterioration by water and oral acids, providing important information on the restoration’s biological safety and durability [23-25]. The release of uncured residual monomers poses a potential sensitizing and irritating factor for oral tissues [26]. Microhardness testing exhibits a strong correlation with spectroscopic procedures and is recognized as a legitimate indirect method for evaluating the degree of cure [27]. Despite limited literature on UFPs and MFPs, both posts are suiTable for wide root canals with extensive coronal destruction. Notably, MFPs involve a two-stage process, with one phase being completed in a laboratory, whereas UFPs only necessitate one session [28]. Another difference is that the cores of UFPs and traditional PFPs form after cement photoactivation, whereas the cores of MFPs may cause light attenuation because core is present before cementation.

The objective of this study was to assess the shear bond strength between CAD/CAM-MFPs, UFPs, and conventional PFPs, as well as the Vickers microhardness of the resin cement when these different posts were used.

We hypothesized that the use of CAD/CAM-milled fiber posts (MFPs) and universal 2-piece fiber posts in widened root canals would result in higher push-out bond strength (BS) and comparable or superior Vickers microhardness values of the self-adhesive resin cement compared to conventional PFPs.

## Material and Methods

The local research ethics committee approved this experiment (protocol number 56948022.7.0000.0105). Thirty sound human mandibular premolars with a minimum root length of 14 mm, free from recent endodontic treatment, caries, fractures, or resorptions, were selected, radiographed, and stored in a 0.1% thymol solution.

The sample size for the push-out test was calculated using pilot data (mean 11.2 MPa, standard deviation 1.7 MPa) with the formula: *n* = Z² x S²/e², where Z = 1.96, the allowed error (e) = 10% of the mean, and S = 1.7 (standard deviation). A minimum sample size of 8.85 was determined, resulting in the random selection of 10 teeth per group. Formal sample size calculations for the secondary outcome (microhardness) were not performed, instead depending on relevant literature references [29].

After decoronation, canals were located with a #10 K-file and shaped using Protaper Ultimate instruments, then irrigated with 2.5% NaOCl, 17% EDTA, and saline. Canals were sealed with AH Plus and gutta-percha using vertical condensation, with glass ionomer cement applied to the cervical area. After 1 week at 37°C, fillings were removed, leaving 4 mm in the apical third. Enlarged canals were standardized with an Exacto drill #3, and the roots were divided into three groups (n = 10): CAD/CAM-milled fiber posts (MFPs), universal 2-piece fiber posts (UFPs), and PFPs.  Table 1 provides a summary of the materials used. All specimens were stored in relative humidity with distilled water for 24 hours at 37°C.

Root canals in each group were prepared to a length of 10 mm using the matching drill to the glass fiber post (Exacto #3; Angelus) connected to the micromotor. A new drill was used after every five root canal preparations. The conduits were irrigated with distilled water using a suction cannula, aspirated, and dried with absorbent paper points.

For the PFP group, fiberglass posts (Exacto No. 3, Angelus) were cemented into the roots, replicating well-adapted fiber posts to the root canals.

**Figure 1:**
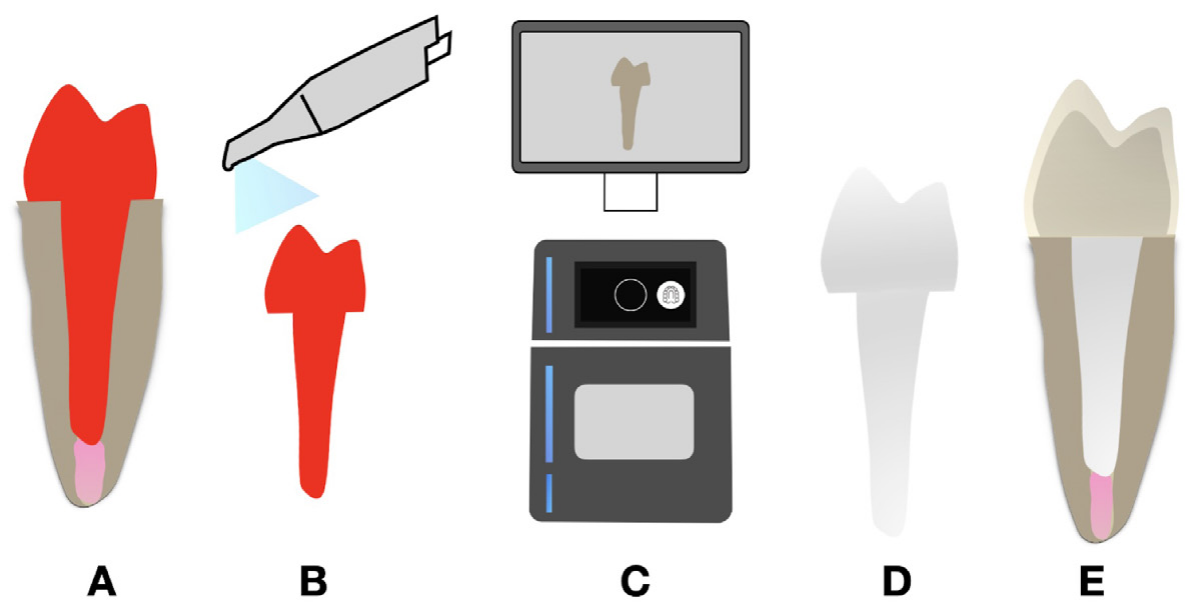
Schematic representative of CAD/CAM milled fiber post. (A) Obtaining the acrylic resin pattern through canal molding with resin pin and acrylic resin; (B) Scanning of acrylic resin pattern; (C) Computer-aided design of the fiber post followed by milling using an industrial computer-aided machine; (D and E) CAD/CAM milled fiber post.

CAD/CAM-MFPs were fabricated as follows. The post space was isolated with aqueous gel and molded by relining a prefabricated acrylic resin pin (Pinjet, Angelus) with acrylic resin (Duralay, Reliance Dental). The acrylic resin posts were then scanned using a digital scanner (TRIOS, 3Shape), and CAD posts were generated based on root dimensions. Glass fiber discs (Fiber Cad - Post & Core, Angelus) were milled using an industrial CAM (Ceramill Motion 2, Amann Girrbach) (Fig. 1).

**Figure 2:**
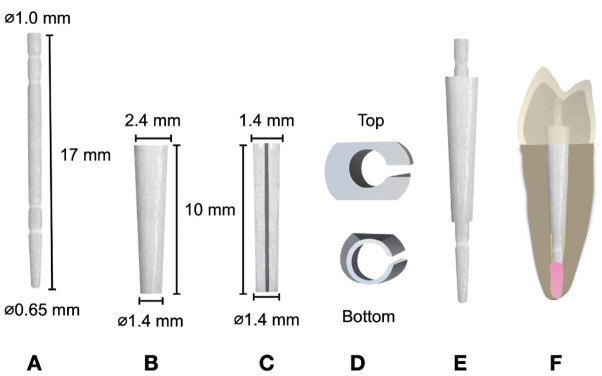
Universal 2-piece post system. (A) Post dimensions; (B) Sleeve front view dimensions; (C) Sleeve lateral view dimensions; (D) Sleeve top and bottom view (E-F) Universal 2-piece post.

The UFP system consists of a universal cylindrical post and a tapered sleeve with the same composition as that of the post. This sleeve features a decreasing thickness from the top (cervical) down to the bottom (apical) end, creating its tapered form, and includes a longitudinal slot that enables it to be adapted into the space between the post and the root canal (Fig. 2).

Before the cementation processes, 70% alcohol was used to clean prefabricated, universal 2-piece (the cylindrical universal post and conical sleeve) and CAD/CAM-MFPs. The posts’ surfaces were coated with silane (Monobond, Ivoclar Vivadent), which was left to dry for 60 seconds. The root canals were then cleaned with distilled water and dried using paper tips.

To perform cementation procedures, all root canals with endo tips were filled with Relyx U200 Automix (Solventum) resin cement, which was subsequently applied to the surface of the fiber posts. To fit between the cervical and middle thirds for the UFP group, the universal cylindrical post was first placed into the canal, and then the tapered sleeve was gradually inserted as far apically as feasible until resistance was encountered. The posts were digitally compressed into the root canals and then slowly and steadily pressed.

With an irradiance of 1,400 W/cm2 (Valo Grand; Ultradent), all fiber posts were light-cured for 60 seconds while being continuously checked with a digital radiometer (LM-1, Woodpecker). Before undergoing the Vickers microhardness (VHN) and push-out BS tests, specimens were kept in distilled water for 24 hours at 37°C.

The roots were then cut into six 1 mm-thick slices perpendicular to their long axis with a low-speed diamond saw (Isomet 1000; Buehler) that was cooled by water. Too much cement was present in the first coronal slice; thus, it was removed. This left six slices per root: two for each of the three coronal sections (cervical, middle, and apical). A 0.01-mm precision digital caliper (Digimatic Caliper) was used to confirm the thickness of each slice, and each slice’s cervical side was recognized. To conduct the push-out BS and VHN experiments, ten roots—the first slice for PBS and the second slice for VHN—were chosen from each group.

Before the push-out test, an optical microscope (BX 51; Olympus) equipped with a digital camera (DP72; Olympus) was used to capture images of each slice from the 10 roots in each experimental group (n = 10) at 40× magnification on both sides. The diameters of the adhesive area (post + cement) in the apical and coronary parts were measured using ImageJ software (National Institutes of Health). The sticky area was calculated using the lateral surface formula of a truncated cone, considering the tapered geometry of the glass fiber post. A universal testing machine (AG-I; Shimadzu Autograph) was used to perform the push-out test, with a crosshead speed of 0.5 mm/min. Every specimen was placed on a metallic apparatus including a central aperture, with its more cervical side pointing downward. A cylindrical metallic tip that matched the post diameter applied a compressive force (a 50-kg load cell) in the apico-coronal direction until debonding. The formula used to calculate the bond strength results was SL=π(R + r)[(h2 + (R – r)2)1/2, where SL is the lateral area of a truncated cone, π = 3.14, R = coronal post + resin cement radius, r = apical post + resin cement radius, and h = thickness of root slice. The formula also considered the maximum force (N) applied.

Following the push-out test, all specimens’ failure modes were examined under 40× magnification optical microscope and categorized as follows: (i) adhesive between the dentin and luting cement; (ii) adhesive between the dentin and post; (iii) cohesive within the dentin; (iv) cohesive within the post; (v) cohesive within the dentin; and (vi) mixed failure.

The second slice of each root third was used to evaluate the microhardness of the resin cement next to the root dentin. The portions were encapsulated in acrylic resin, with the test surface (most coronal region) oriented upward, and polished wet using 600-, 1200-, 1500-, 2000-, and 2500-grit silicon carbide papers (3M), followed by rinsing with tap water. The specimens were preserved for 24 hours at 37 °C. A 100-g force was exerted for 15 seconds during microhardness examination using a Vickers microhardness tester (Shimadzu HMV2, Newage Testing Instruments). Four indentations were created on the self-adhesive resin cement adjacent to the dentin in a clockwise sequence (at 3, 6, 9, and 12 o’clock) on each slice. A minimum distance equivalent to one indentation diameter was preserved from the dentin. The diagonals of each indentation were assessed with an optical microscope at 400× magnification, and the VHN was computed using the formula: VHN = 1.8544 F/d², where 1.8544 is a constant, F represents the force in kgf (0.1 kgf) applied during the test, and d denotes the average of the diagonals of the indentation in mm.

The data from the BS and VHN tests were analyzed by two-way analysis of variance (ANOVA) comparing glass fiber post type and root region, followed by Tukey’s post hoc test (α = 0.05) using Dell Statistica 13.2 software (Dell; Round Rock, TX, USA). The failure mode data were examined solely through qualitative analysis.

## Results

**Figure 3:**
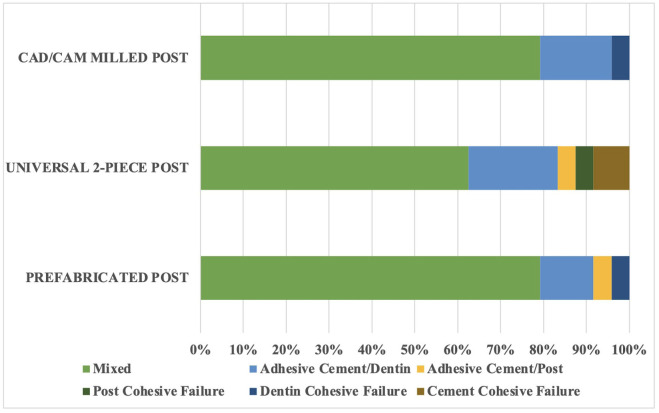
Graph showing the failure mode distribution.

The BS results (mean ± standard deviations) for the different experimental groups are presented in  Table 2. The two-way ANOVA indicated that the cross-product interaction (fiber post type vs. root region) was not significant (*p* > 0.05), with significance observed only for the main factors of root region (*p* < 0.001) and fiber post type (*p* < 0.001). The cervical third exhibited the highest BS values, whereas the apical third showed the lowest values. Among the tested fiber posts, the CAD/CAM-milled (MFPs) and universal posts (UFPs) demonstrated statistically superior BS values compared to the PFPs, with no significant difference between them (*p* > 0.05). The predominant failure mode for all groups was mixed failure (Fig. 3), with cohesive failures occurring less frequently (Fig. 3).

The VHN results (mean ± standard deviations) for the experimental groups are presented in  Table 3. Two-way ANOVA indicated that the cross-product interaction (post type vs. root region) was also not significant (*p* > 0.05). However, it was significant for the main factors of root region (*p* < 0.001) and post type (*p* < 0.001). Regarding root region, the cervical third exhibited the highest VHN values, whereas the apical third displayed the lowest values (*p* < 0,001). Concerning post type, PFPs and UFPs demonstrated the highest VHN values, with no significant differences between UFPs and MFPs (*p* > 0.05), and only a significant difference between PFPs and MFPs (*p* < 0.001).

## Discussion

The restoration of endodontically treated teeth with extensive structural loss remains a significant clinical challenge, necessitating the selection of appropriate intraradicular post systems that ensure favorable long-term outcomes [1]. This study evaluated the influence of CAD/CAM-MFPs, UFPs, and conventional PFPs on the push-out BS and VHN of a self-adhesive resin cement in widened root canals, aiming to contribute to the optimization of restorative strategies in compromised cases.

The results demonstrated that both MFPs and UFPs presented significantly higher BS values than PFPs, corroborating previous findings that anatomically adapted posts enhance the mechanical interlocking and frictional retention within the canal, thereby increasing post retention [5,10,12,14-17]. The superior performance of MFPs and UFPs can be attributed to their improved adaptation to the canal walls, which reduces the thickness of the cement layer and the occurrence of voids, leading to reduced polymerization shrinkage stresses and stress concentration at the adhesive interface [8,10-12,14-17]. Specifically, CAD/CAM technology allows for the fabrication of individualized posts that closely follow the canal anatomy, ensuring close contact with the dentin and a uniformly thin cement layer [14,16]. Similarly, the UFP system, through its adaptable sleeve, ensures a more homogenous fit, decreasing discrepancies within the canal space and improving retention while maintaining simplicity in the clinical workflow [9-12].

In contrast, the use of PFPs in widened canals often results in a thicker resin cement layer due to poor adaptation, which may act as a weak link within the adhesive interface [3,6,8]. The thicker cement layer is more susceptible to polymerization shrinkage and the entrapment of voids, contributing to stress concentration and potential debonding under functional loading [7]. The present findings align with those of Alves dos Santos *et al*. [12], who reported significantly thicker cement layers and larger interfacial gaps in PFPs compared to UFPs, leading to reduced BS values.

The observation that the cervical third exhibited the highest BS across all post systems, whereas the apical third presented the lowest, is consistent with previous studies [19,24,25]. This gradient can be explained by the attenuation of light through the post and resin cement, leading to reduced polymerization efficiency and lower degrees of conversion in the deeper regions of the root canal [20,23-25]. Additionally, the higher density and diameter of dentinal tubules in the cervical third facilitate better micromechanical interlocking of the resin cement, enhancing the adhesive interface [19]. Although self-adhesive resin cements use chemical polymerization to complement light curing, the efficiency of chemical curing alone may not fully compensate for light attenuation, particularly in deeper regions, resulting in reduced mechanical properties such as BS and VHN [20].

The failure mode analysis revealed a predominance of mixed failures across all groups, in agreement with prior studies [10,15,17].This finding suggests that failures primarily occur within the adhesive interfaces rather than within the post or dentin, emphasizing the critical role of interface quality in post retention. The presence of cohesive failures within the post or dentin was minimal, indicating that the mechanical properties of the materials were sufficient to withstand testing forces, and failures were more associated with the quality of the adhesive interfaces and cementation procedures.

Regarding VHN, the PFP and UFP groups exhibited higher values compared to the MFP group, although MFPs demonstrated superior BS. This inverse relationship highlights that although microhardness is an indirect indicator of the degree of conversion, it may not solely dictate the clinical performance of the luting interface [23-25]. The reduced VHN observed with MFPs could be attributable to the attenuation of light by the pre-existing core of the milled post, limiting the depth of cure within the resin cement [23]. Despite this limitation, the optimal adaptation and reduced cement thickness achieved with MFPs likely compensate for the lower VHN, resulting in higher BS values. This finding underscores that achieving intimate post adaptation and minimal cement thickness may have a more significant impact on bond strength than microhardness alone in clinical scenarios involving deep and wide canals.

This study is among the first to evaluate VHN in the cementation of UFP and MFP systems. Although the reduced microhardness in MFPs did not negatively affect BS, the potential long-term implications of lower degrees of conversion, including increased water sorption, solubility, and release of residual monomers, should not be overlooked [23-26]. Further investigations using spectroscopic methods to quantify the degree of conversion alongside microhardness analysis may provide a more comprehensive understanding of the polymerization behavior of resin cements under different post systems and clinical conditions.

Clinically, the findings of this study suggest that CAD/CAM-milled and universal 2-piece fiber posts may be preferable for the restoration of structurally compromised, widened root canals, offering superior mechanical retention without the complexities associated with direct relining procedures using composite resins [5,6]. The CAD/CAM system, while it requires laboratory steps, offers precise anatomical adaptation. In contrast, the UFP system presents a practical, chairside solution that eliminates the need for additional laboratory time, thereby facilitating efficient treatment in cases with severe coronal destruction.

Importantly, this *in vitro* study has limitations, including the absence of long-term aging protocols such as thermocycling or mechanical loading, which could influence the stability of the adhesive interfaces and the degradation of resin cements over time. Future studies incorporating aging protocols, different adhesive strategies, and clinical trials are warranted to validate the long-term performance of MFP and UFP systems in the restoration of endodontically treated teeth with extensive coronal damage.

In summary, this study emphasizes the critical role of post adaptation and cement thickness in optimizing the bond strength of fiber posts within widened root canals. It demonstrates that CAD/CAM-milled and universal 2-piece fiber posts are viable alternatives to conventional PFPs, even when a reduction in cement microhardness is observed.

Despite the higher initial bond strength achieved with CAD/CAM and universal 2-piece fiber posts, future long-term clinical studies incorporating thermomechanical cycling and marginal leakage evaluations are warranted to confirm the stability and clinical performance of these systems in structurally compromised, widened canals.

## Conclusions

CAD/CAM-MFPs and universal 2-piece fiber posts exhibited higher BS results compared to conventional PFPs. Although CAD/CAM-MFPs having lower VHN values, this did not impact their overall performance.

## Figures and Tables

**Table 1:** Composition of the materials used in this study.

Material	Manufacture	Composition
Exacto® Glass Fiber Post #3	Angelus, Londrina, PR, Brasil	Glass Fiber (80%) and epoxy resin (20%)
Splendor®Universal Glass Fiber Post	Angelus, Londrina, PR, Brasil	Glass Fiber (80%) and epoxy resin (20%)
Fiber CAD Post&Core®	Angelus, Londrina, PR, Brasil	Glass Fiber (75-80%) and epoxy resin (20-25%)
RelyX U200	Solventum, St. Paul, MN, USA	Base paste: methacrylate monomers containing phosphoric acid groups, methacrylate monomers, silanated fillers, initiator components, stabilizers, rheological additives. Catalyst paste: methacrylate monomers, alkaline (basic) fillers, silanated fillers, initiator components, stabilizers, pigments and rheological additives.

**Table 2:** Bond strength means and standard deviation (MPa) for the different experimental groups*.

ROOT REGION	EXPERIMENTAL GROUPS (FIBER POSTS)			MAIN FACTOR REGION
	PRE-FABRICATED	UNIVERSAL 2-PIECE	CAD/CAM MILLED	
Coronal	9.2 ± 1.3	11.5 ± 1.7	11.8 ± 2.4	10.8 ± 1.5 A
Medium	6.7 ± 1.5	8.6 ± 2.2	8.5 ± 2.1	7.9 ± 1.1 B
Apical	5.8 ± 2.1	7.2 ± 2.1	7.4 ± 1.7	6.8 ± 1.0 C
MAIN FACTOR POST	7.2 ± 1.8 b	9.1 ± 2.2 a	9.2 ± 2.3 a	

*Different letters indicate statistically significant differences (*p* < 0.001, Tukey’s test).

**Table 3:** Mean microhardness values and standard deviation (VHN) for the different experimental groups*.

ROOT REGION	EXPERIMENTAL GROUPS (FIBER POSTS)			MAIN FACTOR REGION
	PRE-FABRICATED	UNIVERSAL 2-PIECE	CAD/CAM MILLED	
Coronal	74.9 ± 4.3	76.5 ± 3.0	71.4 ± 2.7	74.3 ± 2.6 A
Medium	60.4 ± 3.5	62.4 ± 5.0	59.1 ± 4.4	60.6 ± 1.7 B
Apical	50.7 ± 4.9	53.5 ± 4.2	49.7 ± 4.0	51.3 ± 2.0 C
MAIN FACTOR POST	62.0 ± 12.2 a	64.1 ± 11.6 a	60.1 ± 10.9 b	

*Different letters indicate statistically significant differences (*p* < 0.001, Tukey’s test).

## Data Availability

The datasets used and/or analyzed during the current study are available from the corresponding author.
